# Effects of symmetry-breaking on electromagnetic backscattering

**DOI:** 10.1038/s41598-020-80347-5

**Published:** 2021-01-18

**Authors:** Mohamed Ismail Abdelrahman, Evgeniia Slivina, Carsten Rockstuhl, Ivan Fernandez-Corbaton

**Affiliations:** 1 Institute of Theoretical Solid State Physics, Karlsruhe Institute of Technology, 76131 Karlsruhe, Germany; 2 Aix Marseille Univ, CNRS, Centrale Marseille, Institut Fresnel, 13013 Marseille, France; 3 Institute of Nanotechnology, Karlsruhe Institute of Technology, 76021 Karlsruhe, Germany; 4 Meyer Burger Research AG, 2068 Hauterive, Switzerland; 5 Present Address: School of Electrical and Computer Engineering, Cornell University, Ithaca, NY 14853 USA

**Keywords:** Nanophotonics and plasmonics, Sub-wavelength optics

## Abstract

Systems with a discrete rotational symmetry $2\pi /n$ where $n\ge 3$ that also have electromagnetic duality symmetry exhibit zero backscattering. The impact of breaking one of the two symmetries on the emerging backscattering has not yet been systematically studied. Here, we investigate the effect that perturbatively breaking each of the two symmetries has on the backscattering off individual objects and 2D arrays. We find that the backscattering off electromagnetically-small prisms increases with the parameters that determine the symmetry breaking, and that the increase of the backscattering due to the progressive breaking of one of the symmetries can be related to the other symmetry. Further exploration of the interplay between the two symmetries reveals that, in systems lacking enough rotational symmetry, the backscattering can be almost-entirely suppressed for a given linear polarization by deliberately breaking the duality symmetry. This duality breaking can be interpreted as an effective increase of the electromagnetic degree of rotational symmetry for that linear polarization.

The seminal work by Kerker et al.^1^ on backscattering suppression has been recently understood as a consequence of systems having two symmetries: Discrete rotational symmetry and duality symmetry^2–5^. In Ref.^5^ two sufficient conditions for the complete suppression of backscattering from a general system are identified: (i) A discrete rotational symmetry, from the perspective of the illuminating plane wave, of at least third order $n \ge 3$, so $C_3$, $C_4$, etc, including the cylindrical symmetry as the limiting case $C_\infty$, and (ii) helicity preservation, that is, zero cross-talk between the two polarization handedness of the field upon light-matter interaction (see Fig. 1). The symmetry that achieves helicity preservation in a geometry-independent way is the electromagnetic duality symmetry. Very recent work^6^ shows that separately meeting each of the two symmetry conditions, duality and $C_{n\ge 3}$, is not necessary: Invariance of the system under simultaneous discrete geometric and duality rotations with the same angle $\theta =\frac{2\pi }{n}$ for $n\ge 3$ is sufficient for achieving zero backscattering (ZBS).

For the macroscopic Maxwell equations, a system composed piece-wise by domains of homogeneous and isotropic materials is dual symmetric if and only if $\frac{\mu _i}{\varepsilon _i}$ is constant throughout the domains^4^ (see Fig. 1). We note that helicity preservation under particular illumination conditions can be achieved by geometrical optimization for systems where $\varepsilon _\text {r}\ne \mu _\text {r}=1$, allowing the theoretical consideration and design^6–20^ of systems with sufficient conditions for ZBS. Such systems can be practically realized^8,21–25^ and have potential applications in holography and anti-reflective nano-coatings for ultra-thin solar cells.

**Figure 1:**
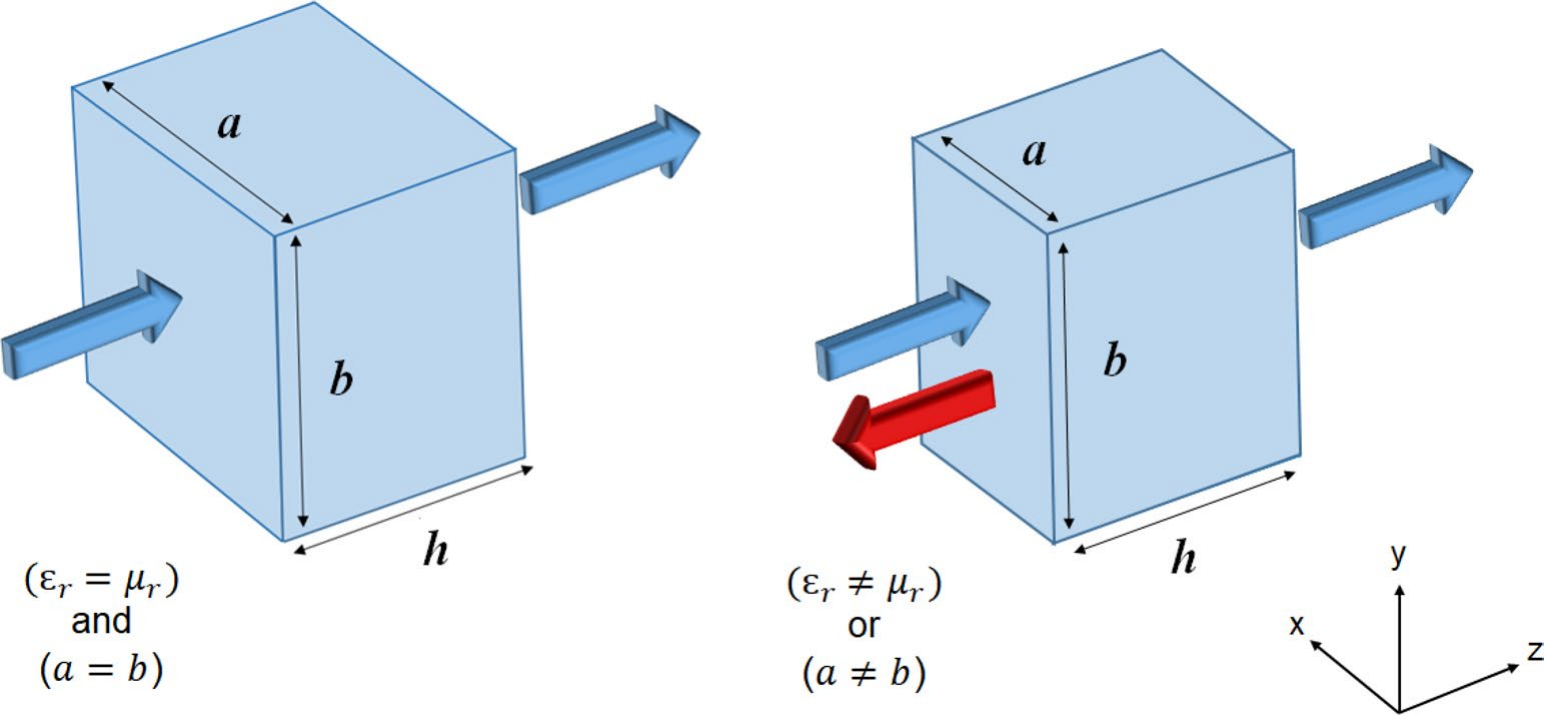
Two conditions that, together, are sufficient for ZBS: A rectangular prism of height *h* illuminated by a normally incident plane wave (propagating in the $+z$-direction) of an arbitrarily polarization exhibits ZBS when the prism base sides are equal $a=b$ ($C_4$ symmetry) and the prism material is dual $\varepsilon _\text {r}=\mu _\text {r}$ at the excitation frequency.

In general, ZBS behavior has been extensively investigated in the literature for symmetrical systems. In contrast, the behavior of systems with broken symmetry conditions is less explored. Our purpose here is to systematically investigate the effect of perturbatively breaking the ZBS symmetry conditions. We will consider $\mu _\text {r} \ne 1$ materials for the straightforward implementation of duality symmetry and its breaking. While the results are hence not directly applicable in many frequency bands, e.g. at optical frequencies, this approach allows us to treat duality symmetry in a geometry-independent way.

In this article we investigate the effect that breaking the ZBS symmetry conditions has on the backscattering off electromagnetically-small (dipolar-regime) prisms, individually, and in 2D arrays. We first consider systems with sufficient rotational symmetry and examine the backscattering as the material parameters deviate from the duality point $\varepsilon _\text {r}/\mu _\text {r}=1$. For single prisms we find that, for a fixed value of $\varepsilon _\text {r}/\mu _\text {r}\ne 1$, the backscattering decreases monotonically as the degree of rotational symmetry increases, with cylinders showing the least backscattering. For 2D arrays of prisms, the interplay between lattice and prism symmetry gives rise to a richer set of effects. In the immediate vicinity of the duality point, the backscattering decreases monotonically as the overall degree of rotational symmetry of the system increases. For large deviations from the duality point the backscattering decreases monotonically as the prism symmetry increases. In between these two regimes, there is an intermediate region where the backscattering is not ordered according to a single symmetry number. For arrays with insufficient degree of rotational symmetry ($n=\{1,2\}$), the backscattering at the duality point decreases monotonically with *n* and also with the degree of prism symmetry *n*. We then consider dual-symmetric systems where the initially sufficient rotational symmetry is *increasingly* broken into $C_2$: A single sphere deformed into an ellipsoid, and a 2D-array of disks whose initial square lattice is deformed into a rectangular lattice. The two systems show the same behavior: The larger the parameter that controls the symmetry breaking, the larger the backscattering, which is almost equal for similar deformations along different orthogonal axes. Additionally, we study how, for the particular linear polarization (LP) that is adapted to the geometrical symmetry breaking, the backscattering can be minimized to values comparable to those numerically achieved by ZBS systems^26,27^. The minimization is achieved by compensating the rectangular anisotropy with a permittivity to permeability ratio different from unity. The compensation can be seen as the restoration of an effective electromagnetic $C_4$ symmetry for the geometrically $C_2$ ellipsoid(array), *for that particular linear polarization*. For electromagnetically small ellipsoids, the ratio of material parameters that minimizes the LP backscattering for a given axial ratio can be predicted analytically to a very good approximation.

## Results

### Backscattering (reflection) off individual objects and 2D arrays

We start by studying the backscattering off isolated prisms. We consider different *n*-sided prisms (featuring $C_{n}$ symmetry), namely: Triangular $n=3$, square ${n}=4$, hexagon ${n}=6$, and a cylinder ${n} \rightarrow \infty$. All the discrete objects that we consider throughout the article have a volume *V* equal to the volume of a sphere of one-tenth-the-wavelength radius and a height (extend in *z*) $h=\root 3 \of {V}$. The illumination is a RCP plane wave propagating in the direction parallel to the axis of symmetry of the prisms (*z*-axis). It is important to note that, for systems with ${n} \ge 3$ that additionally have a mirror symmetry across a plane containing the optical axis, the backscattered power is independent of the polarization: The particular choice of RCP does not imply any loss of generality. We will consider the backscattering ratio $R_\text {b}=Q_\text {b} / Q_\text {sca}$, where the backscattering efficiency $Q_\text {b}$ and the scattering efficiency $Q_\text {sca}$ are defined in Ref.^28^ (Secs. (4.6) and (3.4), respectively). For spherical particles, both quantities can be directly evaluated using Mie coefficients, while in the general case numerical calculations are necessary. The quantity $Q_\text {b}$ is proportional to the absolute value square of the far-field amplitude of the scattered electric field in the backward direction relative to the illumination. The far-field scattered electric field is defined as $\mathbf{E }_\text {far}(\theta ,\phi ) = \lim _{r\rightarrow \infty } \, r \, \mathbf{E }_\text {sca}(r,\theta ,\phi )$, where $(r,\theta ,\phi )$ are the radial, polar, and azimuthal spherical coordinates. For lossless particles like the ones we consider throughout the article, the optical theorem can be used to evaluate $Q_\text {sca}$ [28, Eq. (3.24),]: $Q_\text {sca}$ is proportional to the imaginary part of the complex dot product between the total far-field $\mathbf{E }_\text {far}$ in the forward direction $(\theta =0,\phi =0)$ and the polarization vector of the incident field. For example, for a RCP incident plane wave with electric field given by $\mathbf{E }_\text {i}= e^{i k_\text {0} z}\,({\hat{x}}-i {\hat{y}})$, the backscatttering ratio can be computed as

1 $$R_{{\text{b}}} = \sqrt 2 k_{0} \frac{{\left| {{\mathbf{E}}_{{{\text{far}}}} (\theta = \pi ,\phi = 0)} \right|^{2} }}{{{\text{Imag}}\left\{ {E_{{{\text{far\_x}}}} (\theta = 0,\phi = 0) + iE_{{{\text{far\_y}}}} (\theta = 0,\phi = 0)} \right\}}}.$$

**Figure 2:**
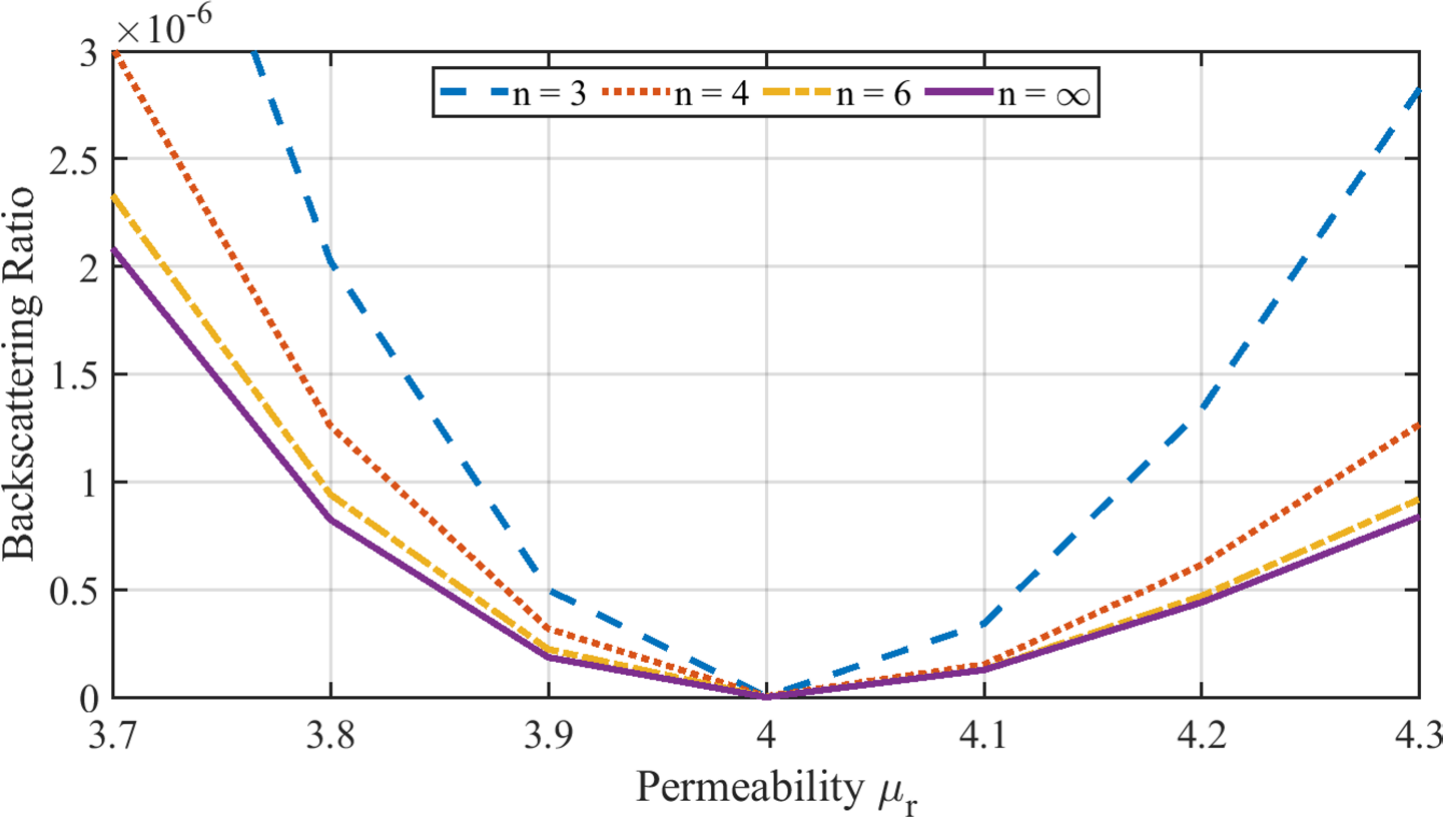
Backscattering ratio $R_\text {b}$ of different *n*-sided prisms (legend) with permittivity $\varepsilon _\text {r}=4$ as a function of permeability around the duality point $\varepsilon _\text {r}=\mu _r$.

Figure 2 shows $R_\text {b}$ as a function of the permeability of the prisms around a fixed permittivity $\varepsilon _\text {r}=4$. We observe that the backscattering increases as $\varepsilon _\text {r}/\mu _\text {r}$ departs from the duality point $\varepsilon _\text {r}/\mu _\text {r}=1$, and that the higher the degree of discrete rotational symmetry, the smaller the backscattering for a fixed value of $\varepsilon _\text {r}/\mu _\text {r}\ne 1$, with the cylinder exhibiting the least backscattering. The behavior of $R_\text {b}$ is smooth near the duality point and decreases monotonically while approaching it, consistently with the findings in Ref.^11^ for the case of spherical particles.

Since symmetry conditions for ZBS apply to general systems, we now study the reflection off periodic planar arrays of identical prisms. We consider square and hexagonal 2D Bravais lattices. The reflection coefficient *R* is defined as the reflected power divided by the input power. The reflected power is calculated as the integral over the surface of a unit cell of the component of the Poynting vector on the backward direction, opposite to the illumination.

**Figure 3:**
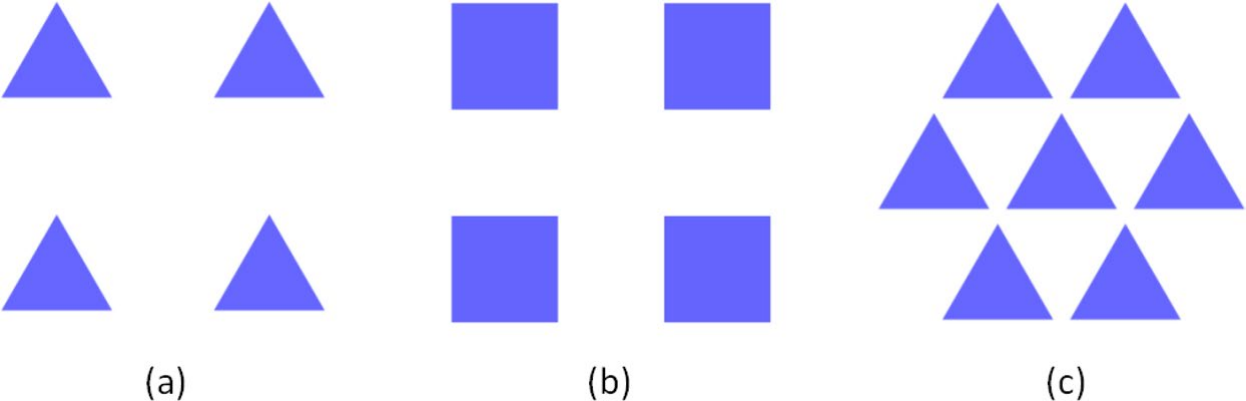
(**a**) An array of triangular prisms ($n_p=3$) arranged in a square lattice ($n_l=4$) will not be mapped onto itself if it is rotated by any angle rather than multiples of $360^{\circ }$ (overall symmetry $n=1$), while (**b**) an array of square prisms ($n_p=4$) arranged in a square lattice ($n_l=4$) is symmetric under rotations by multiples of $90^{\circ }$ (overall symmetry $n=4$), and (**c**) an array of triangular prisms ($n_p=3$) arranged in a hexagonal lattice ($n_l=6$) is symmetric under rotations by multiples of $120^{\circ }$ (overall symmetry $n=3$).

In the following, we adopt the notation $[n_p,n_l,{n}]$ to label a given choice of $n_p$-sided prisms, lattice symmetry $n_l\in \{4,6\}$, and overall degree of discrete rotational symmetry of the system *n*, which depends on both $n_p$ and $n_l$. For example, triangular prisms ($n_p=3$) arranged in a square lattice ($n_l=4$) result in a combined system without rotational symmetry, hence ${n}=1$. Table 1 shows the considered $[n_p,n_l,{n}]$ combinations, while Fig. 3 illustrates a few examples pictorially.

**Table 1:** The combinations of prisms, lattices, and the resulting overall degree of discrete rotational symmetry *n* for all the arrays that we consider.

$n_p$-sided prisms	3	4	6	$\infty$	3	4	6	$\infty$
lattice $n_l$	4	4	4	4	6	6	6	6
overall symmetry ${n}$	1	4	2	4	3	2	6	6

**Figure 4:**
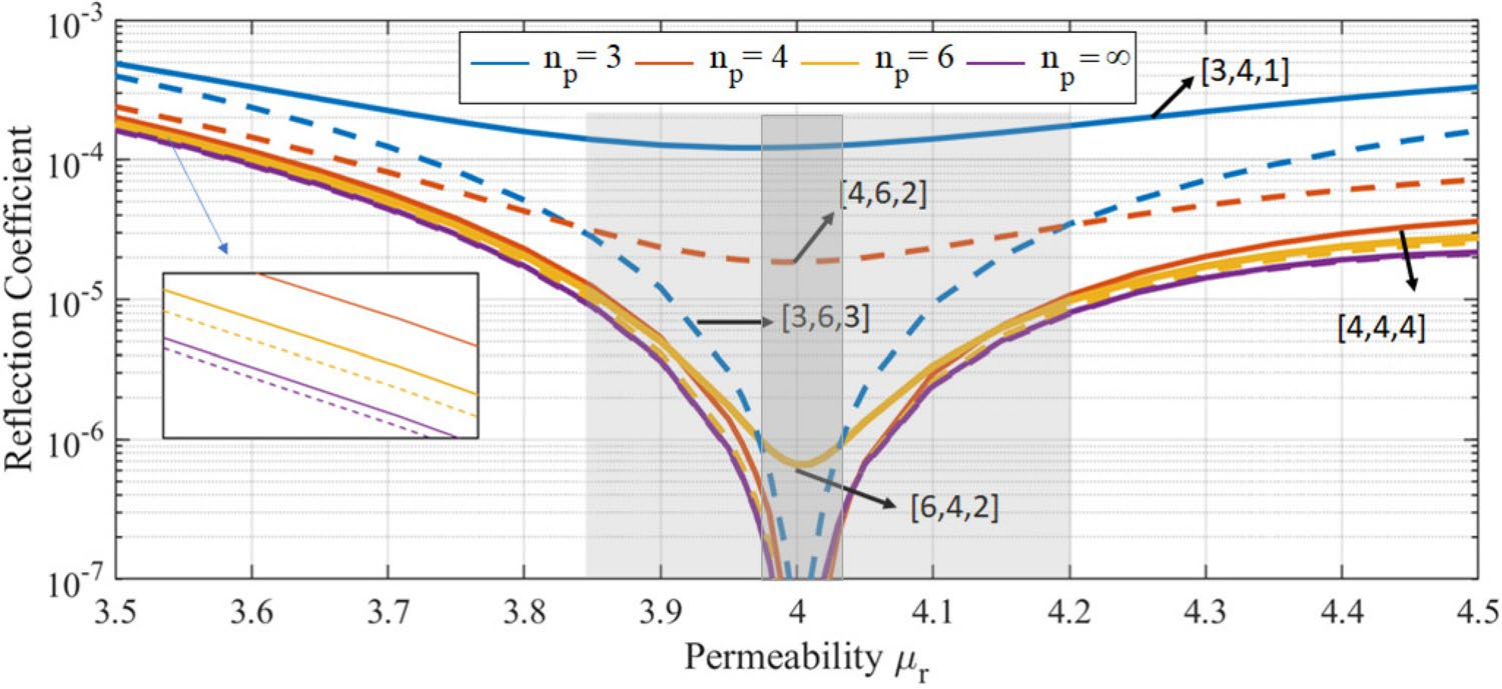
Reflection of square (solid) and hexagonal (dashed) lattices of identical $n_p$-sided prisms (legend: $n_p$-order) as a function of the permeability. The permittivity is fixed at $\varepsilon _\text {r}=4$. The labels $[n_p,n_l,n]$ indicate the rotational symmetry of the prisms ($n_p$), the lattice $(n_l)$, and the overall system (*n*). The lattice pitch is equal to $26\%(28\%)$ of the wavelength for square(hexagonal) lattices.

The reflection coefficient for square (solid lines) and hexagonal (dashed lines) lattices of identical prisms with different *n*-fold symmetry is shown in Fig. 4 as a function of the permeability of the prisms around a fixed permittivity $\varepsilon _\text {r}=4$. The portion of unit cell area occupied by the prisms is the same in both kinds of lattices. This is achieved by setting the lattice pitch to $26\%$ of the excitation wavelength for the square lattices and $28\%$ for the hexagonal lattices. Only a zeroth diffraction order occurs in all cases.

Let us first analyze the behavior at the duality point in Fig. 4. We have removed the data points for $\varepsilon _\text {r}=\mu _\text {r}$ for some configurations because their very small values are detrimental to the visibility of the rest of the data. As expected, all the systems with ${n}\ge 3$, like square lattices of square prisms [4, 4, 4] or cylinders $[\infty ,4,{4}]$, and hexagonal lattices of triangular prisms [3, 6, 3], hexagonal prisms [6, 6, 6], or cylinders $[\infty ,6,{6}]$, exhibit ZBS at the duality point $\varepsilon _\text {r}=\mu _\text {r}$ up to numerical accuracy. On the other hand, the configurations with ${n}<3$ do not achieve ZBS at the duality point. For these ${n}<3$ cases, we observe that the reflection at the central point decreases monotonically when ${n}(n_p)$ increases. As we move away from the duality point we can identify an interval where the backscattering of the ${n}\ge 3$ systems keeps below that of the ${n}<3$ systems. We will call this interval the perturbation region, which is marked by a dark gray background. In this perturbation region the backscattering decreases monotonically with *n*. Immediately outside the perturbation region, the light gray background marks an intermediate region where the backscattering of the different systems is not ordered by a single number. For an even larger $\varepsilon _\text {r}$ and $\mu _\text {r}$ mismatch, the backscattering decreases monotonically as the rotational symmetry of the prism ($n_p$) increases (see the inset). We note that the width of both near-duality perturbation region and intermediate region shrink when the lattice pitch to wavelength ratio increases, as observed in the results of extra simulations (not included here) where a ratio of $35\%$ was used. As the particle separation increases, the electromagnetic coupling between the particles in the array decreases which, at its turn, decreases the relevance of the lattice symmetry, and shrinks both regions. In the limit of large separation, the overall symmetry *n* is effectively determined by the prisms symmetry $n_p$ and the systems should behave as dictated by the latter (see Fig. 2), which is what we observe in the results. Finally, we note that for $n\ge 3$ systems made out of the same prisms, the hexagonal lattice results in smaller backscattering than the square lattice. This behavior has been reported for fully dielectric $\mu _\text {r}=1$ systems^18^.

**Figure 5:**
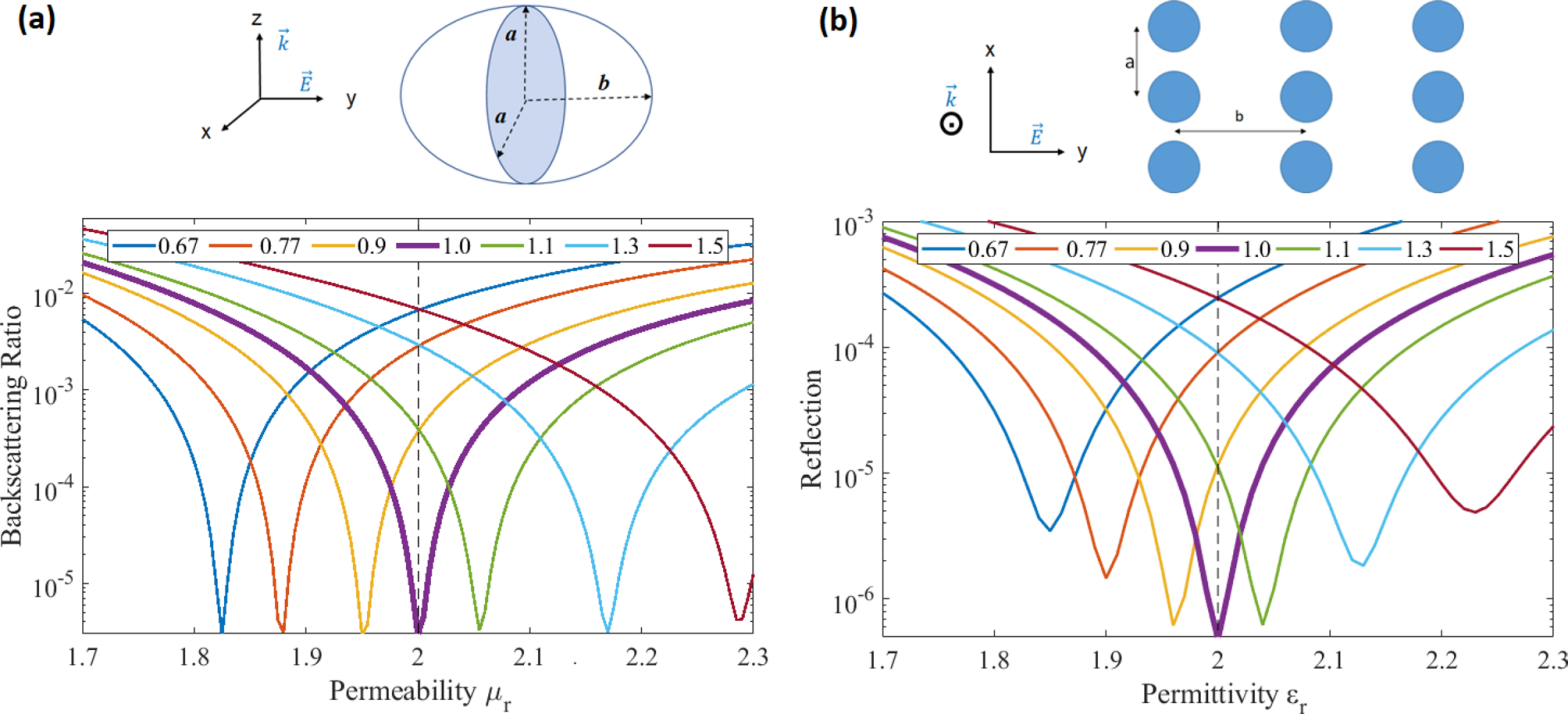
(**a**) Backscattering ratio $R_\text {b}$ for a ellipsoid of permittivity $\varepsilon _\text {r}=2$ for a varying axial ratio *b*/*a* (legend) as a function of the permeability. (**b**) Reflection off a 2D array of identical cylinders of permeability $\mu _\text {r}=2$ arranged in a rectangular lattice for varying pitch ratio (legend) of the lattice as a function of the permittivity. The top parts of each figure show the geometries and illuminations. The electric field of the illumination is parallel to the distinct axis of the ellipsoid (of length *b*), and to the lattice vector whose length is the numerator of the pitch ratio of the array. The horizontal dashed lines mark the duality points $\varepsilon _\text {r}/\mu _\text {r}=1$.

We now investigate the backscattering off $\varepsilon _\text {r}=\mu _\text {r}$ dual systems as the initially sufficient degree of rotational symmetry for ZBS is *progressively* broken into $C_2$. We consider two systems: A single sphere deformed into an ellipsoid of axes (*a*, *b*, *a*) as the axial ratio *b*/*a* deviates from unity, and a 2D-array of disks whose initial square lattice is deformed into a rectangular lattice breaking ${n}=4$ into ${n}=2$. While in general the backscattered power off $C_2$ systems depends on the polarization, it is straightforward to show that the backscattered power off dual-symmetric systems with a mirror symmetry across a plane containing the optical axis does not depend on the incident polarization. Therefore, without loss of generality, we can choose a particular linear polarization which is adapted to the system, and to the geometrical symmetry breaking. Namely, the electric field of the incident plane wave is linearly polarized along the *y*-axis, which coincides with the *b* dimension of the ellipsoid and with one of the two lattice vectors of the array. The plane wave propagates along the *z*-axis, perpendicularly to the plane of the array. Only the main diffraction order is allowed in all the different array lattices. Figure 5 shows the results. The backscattering at the duality point $\varepsilon _\text {r}/\mu _\text {r}$ in Fig. 5a,b shows that: (i) The further the axial(pitch) ratio deviates from unity (The larger the $C_\infty (C_4)$ symmetry breaking), the larger the backscattering, and, notably that (ii) axial(pitch) ratios that correspond to the same degree of symmetry breaking like 0.9 and 1.1, or 0.77 and 1.3 exhibit almost the exact same backscattering at the duality point. This observation can be related to previous ones: It is similar to the decrease of backscattering as the rotational symmetry increases in systems with ${n}<3$ at the duality point, and, by switching the roles of duality and rotational symmetries, can be seen as the counterpart of the increase of backscattering as the duality symmetry of a system with sufficient discrete rotational symmetry is increasingly broken.

### Backscattering suppression for systems of a broken symmetry

**Figure 6:**
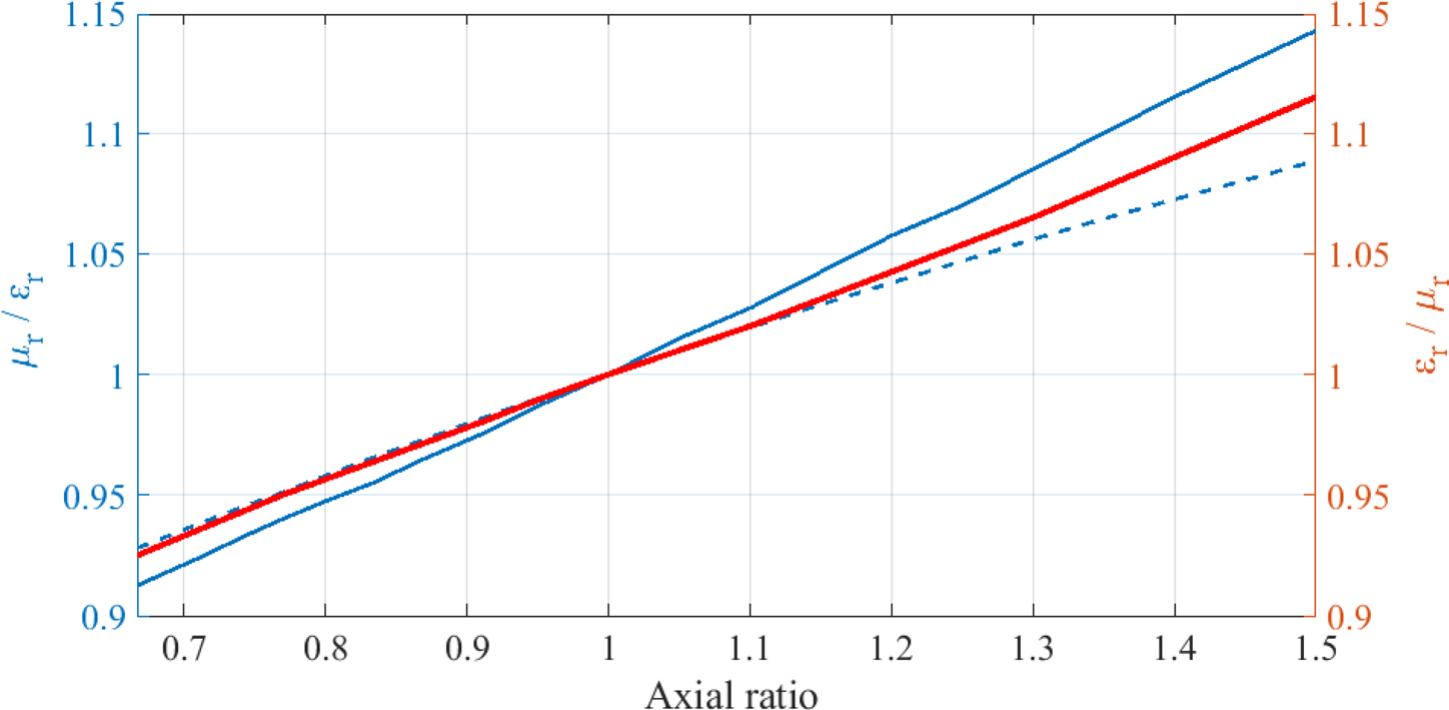
The permeability to permittivity(permittivity to permeability) ratio that achieves minimum backscattering for each different axial(pitch) ratio of the ellipsoid(array) is shown by the continuous blue(red) line. The blue dashed line is an analytical prediction of the axial ratio required for the ellipsoid.

Figure 5 also shows that the backscattering for the chosen linear polarization is minimized for some ratio of the material parameters away from the duality point. This is a remarkable feature as it suggests that the breaking of rotational symmetry can be compensated by deliberately breaking the duality symmetry to restore the vanishing of backscattering. We now investigate this further. However, it is important to keep in mind that: (i) Such minimization is not equivalent to ZBS, which implies zero back reflection *for all incident polarizations*, and that (ii) the backscattering off mirror symmetric systems is independent of the polarization only at the duality point. Away from duality, the dips in Fig. 5 are reflection minima for the chosen linear polarization. Figure 6 shows the permeability to permittivity (permittivity to permeability) ratio that achieves minimum backscattering for each different axial (pitch) ratio of the ellipsoid (array). This graph, and in particular, the inversion of the material parameters ratio from the ellipsoid to the array can be understood as follows. We consider the strength of the response of the systems in the direction of the electric field relative to the strength of the response of the systems in the direction of the magnetic field. Such relative strength grows as the axial ratio of the single ellipsoid increases because the induced electric dipole moment grows with respect to the induced magnetic dipole moment. On the other hand, the relative strength decreases as the pitch ratio of the rectangular cell of the 2D array increases because the array becomes less dense along the electric field direction relative to its density along the magnetic field direction. Then, for the materials to compensate the geometrically induced change in the relative strength, $\mu _\text {r}/\varepsilon _\text {r}$ must increase in the case of the ellipsoid and decrease in the case of the array. Such compensation can be interpreted as the induction of an effective electromagnetic $C_4$ symmetry onto the geometrically $C_2$-symmetric ellipsoid(array), *but only for the particular linear polarization*.

Finally, we will show that the $\mu _\text {r}/\varepsilon _\text {r}$ ratio that achieves the minimal backscattering off the ellipsoid for the chosen linear polarization can be predicted analytically to good approximation when considering the electric and magnetic dipole polarizability of a ellipsoid and ignoring higher multipolar orders. This is a reasonable approximation because the considered ellipsoid has a volume corresponding to a sphere with radius equal to one-tenth of the excitation wavelength. The normalized electric dipole polarizability of a ellipsoid is anisotropic and is given by Ref.^29^ as

2 $$\begin{aligned} (\alpha _\text {e})_i= \dfrac{\varepsilon _\text {r}-1}{1+ N_i (\varepsilon _\text {r}-1)}, \end{aligned}$$

where *i* represents the component of the polarizability along the axes *x*, *y*, *z*. $N_i$ is the depolarization factor of the ellipsoid in the *x*, *y*, *z*-directions, which can be calculated numerically depending on the ellipsoid axial ratio, and $N_x+N_y+N_z=1$. The normalized magnetic dipole polarizability can be inferred using a duality transformation,

3 $$\begin{aligned} (\alpha _\text {m})_i= \dfrac{\mu _\text {r}-1}{1+ N_i (\mu _\text {r}-1)}. \end{aligned}$$

For a nondual material of the ellipsoid ($\varepsilon _\text {r} \ne \mu _\text {r}$), minimum backscattering is anticipated when the electric dipole polarizability equals its magnetic counterpart. The condition of minimum linearly polarized backscattering for an incident *y*-polarized plane wave can be easily derived to be:

4 $$\begin{aligned} (\alpha _\text {e})_y=(\alpha _\text {m})_x. \end{aligned}$$

Equations (2–4) allow to compute the axial ratio that achieves Eq. (4) for a particular permeability to permittivity ratio. The analytical prediction is shown by the blue dashed line in Fig. 6, and is in very good agreement with the actual optimal axial ratio values extracted from the simulations (continuous blue dashed line). The previous interpretation of electromagnetic $C_4$ restoration onto a geometrical $C_2$ system by means of $\mu _\text {r}/\varepsilon _\text {r}\ne 1$ can be appreciated in the fact that Eq. (4) is met by a $C_4$ system with $\mu _\text {r}/\varepsilon _\text {r}=1$.

In conclusion, we have analyzed the consequences of perturbatively breaking the two symmetries involved in zero-backscattering: Discrete rotational symmetry $C_{n\ge 3}$, and duality symmetry. In general terms, when one of the symmetries is broken, the backscattering grows with the parameter that controls the symmetry breaking, and very similar behavior can be observed in both single objects and regular 2D arrays. For isolated $C_{n\ge 3}$-symmetric prisms, the growth of the backscattering as duality is increasingly broken is slower for larger values of *n*, and the cylinder with $n\rightarrow \infty$ shows always the least backscattering. A similar, albeit not identical behavior can be observed in 2D arrays, where both the inclusion and lattice symmetries play a role. For dual systems, the progressive breaking of the sufficient discrete rotational symmetry results in a progressive increase of the backscattering in both isolated objects and arrays. Finally, it is seen that the violation of the sufficient degree of rotational symmetry in the ellipsoids and $C_2$ arrays can be compensated for a particular linearly-polarized illumination by a corresponding deliberate breaking of the duality symmetry. We consider the comparison of these results with a study of symmetry-breaking in $\varepsilon _\text {r}\ne \mu _\text {r}=1$ systems where helicity preservation is achieved by geometrical and material optimization to be a worthwhile future step. We also consider to extend the investigation of symmetry-breaking effect on backscattering to larger objects, comparable to the excitation wavelength, which could potentially enhance our understanding of the intricate backscattering behaviour of complex scattering systems.

## Methods

COMSOL Multiphysics^®^ (version 5.2, COMSOL AB, Stockholm, Sweden) and JCMsuite 3.18^30^ are used for numerical calculation of the far-fields. The perfectly matched layer (PML) approach is implemented to truncate the far-field problem onto a shell surrounding the scattering particle (see Ref.^31^). The meshing of particles is critical especially when the effect of rotational symmetry is investigated. The tetrahedron-type meshing used typically breaks rotational symmetry of particles, unless specifically designed to avoid it. Alternatively, the meshing has to be fine enough so that symmetry breaking due to the meshing process is negligible. Therefore, multiple tools were used to confirm results.
